# Surgical Management of Primary Nasal Mucosal Melanoma: A Case Report

**DOI:** 10.7759/cureus.87422

**Published:** 2025-07-07

**Authors:** Vaia Karapepera, Dimitrios Tziouris

**Affiliations:** 1 Ear, Nose, and Throat Clinic, General Hospital of Ioannina G. Hatzikosta, Ioannina, GRC; 2 Otolaryngology-Head and Neck Surgery, General Hospital of Ioannina G. Hatzikosta, Ioannina, GRC

**Keywords:** melanoma, mucosal, mucosal melanoma, nasal melanoma, sinonasal

## Abstract

Primary sinonasal mucosal melanoma (PSMM) is a rare and aggressive malignancy of the nasal cavity and paranasal sinuses. PSMM presents significant diagnostic and therapeutic challenges due to its nonspecific symptoms, complex anatomy, and high recurrence rate. Due to its vague symptoms, diagnosis is often delayed, leading to poor outcomes. Early detection and prompt surgical intervention are crucial for improving prognosis. Surgical excision with clear margins remains the primary treatment, while the role of adjuvant therapies continues to develop. We present the case of a 56-year-old male patient with progressive left-sided nasal obstruction, intermittent epistaxis, and anosmia. Nasal endoscopy revealed a pigmented mass in the left nasal cavity. Histopathological examination and immunohistochemistry confirmed the diagnosis of mucosal melanoma. The patient underwent endoscopic surgical resection followed by adjuvant radiotherapy, with no postoperative complications. He remains under close follow-up. This report aims to highlight the surgical approach to a rare case of PSMM. This case highlights the importance of early detection and intervention and underscores the need for continued case reporting to inform future management approaches.

## Introduction

Primary sinonasal mucosal melanomas (PSMMs) constitute rare and aggressive malignancies, deriving from melanocytes of the nasal mucosa. Only 0.2%-0.5% of all melanomas occur in the nasal cavity and paranasal sinuses, and they comprise less than 5% of all sinonasal malignancies [1,2]. These cancers appear more frequently in men than women (2:1 ratio), in the Caucasian population than in the African American population, and more commonly present over the age of 60 [1,3]. Interestingly, mucosal melanomas tend to exhibit more aggressive behavior than their cutaneous counterparts. This is primarily because their location makes early detection more difficult, often leading to a diagnosis at an advanced stage compared to cutaneous melanoma, which appears on the skin and is more easily noticeable [1-3]. The most common symptoms of nasal mucosal melanomas (NMMs) include recurrent epistaxis, nasal congestion or obstruction (most often unilateral), rhinorrhea, pain, and headaches. As the disease progresses, more symptoms may arise, such as hyposmia, deformity of the nose, and diplopia and proptosis [1,2]. However, these clinical signs may manifest gradually, thus making the tumor less likely to be diagnosed at an early stage, and this in turn negatively impacts the patient’s survival [1,2]. In addition to that, NMMs tend to have a significant risk for local recurrence (31%-85%) as well as distant metastases (25%-50%) [4]. The five-year survival is usually less than 30% and ranges from 20% to 50% [1,5]. While the median time to relapse is approximately one year or less, late recurrences are frequently observed, and disease-specific survival continues to decrease beyond the five-year mark [4]. Diagnosis is established by histological and immunohistochemical results. We report a case of a 75-year-old male patient with NMM, presenting with epistaxis, to highlight the challenges in early diagnosis and management of this rare malignancy.

## Case presentation

**Figure 1:**
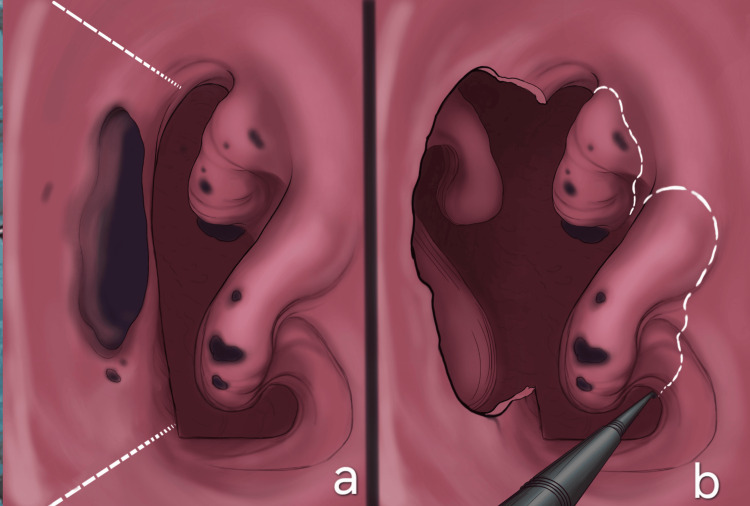
(a, b) Surgical excision Created by the author Vaia Karapepera

**Figure 2:**
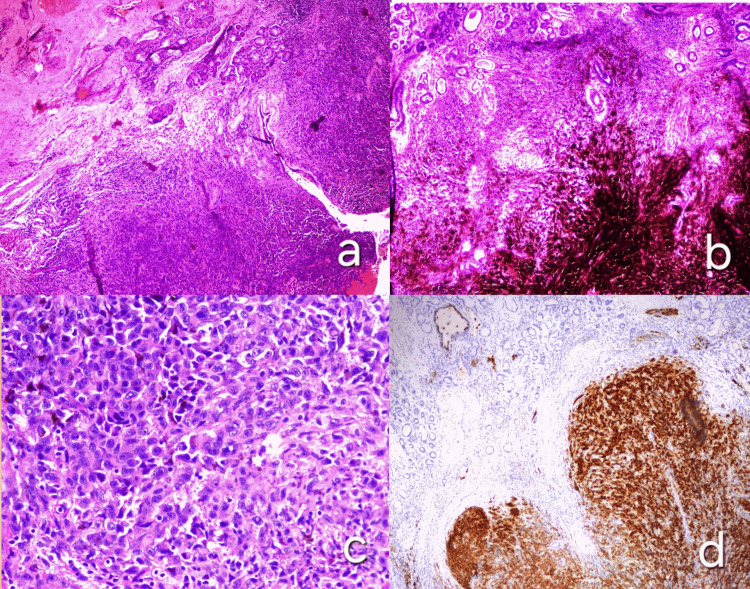
Histopathology (a) Hematoxylin and eosin stain showing melanoma cell infiltration in the nasal mucosa (10x magnification). (b) Melanin pigment deposition within the tumor cells (10x magnification). (c) Epithelioid melanoma cells with eosinophilic cytoplasm and prominent nucleoli (40x magnification). (d) Immunohistochemistry demonstrating positive HMB45 staining in tumor cells (10x magnification)

A 75-year-old male patient presented to our clinic with frequent spontaneous nosebleeds, unilateral nasal congestion, and nasal pain for 12 months. He reported that symptoms did not improve with standard treatments for nasal congestion and were gradually progressing over the course of these months. His medical history included hypertension, dyslipidemia, and chronic obstructive pulmonary disease (COPD). Also notable is his 40-year history of heavy smoking, consuming two packs per day. An endoscopy examination of the nasal cavities was performed, which revealed a solid darkly pigmented mass occupying most of the septum surface and several smaller dot-like lesions of similar color in the neighboring inferior and middle nasal conchae (Figure 1). An incisional biopsy was conducted, which established the diagnosis of malignant melanoma. Specifically, histological examination (using hematoxylin and eosin stains) revealed that the mucosa of the nasal cavity was infiltrated by melanoma cells (Figure 2a), with abundant melanin pigment depositions (Figure 2b). The infiltrative population was composed of epithelioid cells with eosinophilic cytoplasm and large nuclei with prominent nucleoli (Figure 2c), positive for HMB45 immunostaining (Figure 2d). Further endoscopic examination depicted the neoplasm measuring 55 × 40 × 50 mm, which occupied and eroded a large part of the nasal septum and vomer. The mass did not erode or invade the base of the skull, nor did it extend intracranially. Regular hematological and biochemical examinations were within normal limits. Chest and neck CT scans revealed no regional lymphadenopathy and no evidence of metastatic lesions in the lung or mediastinum. Thus, the patient was staged as cT4aN0M0.

The patient underwent endoscopic endonasal surgery under general anesthesia. As the carcinoma extended posteriorly toward the anterior wall of the sphenoid sinus, an extensive excision of the septum and vomer was performed using monopolar diathermy and cold steel instruments. Endoscopic type D medial maxillectomy was performed. Specifically, the lower turbinate was excised en bloc with the medial wall of the maxilla, and the middle turbinate was removed up to the skull base in order to further achieve clear surgical margins (20 mm), ensuring complete removal of the malignancy (Figure 1). All removed tissues were submitted for histological examination. Postoperative recovery was uneventful; the patient was discharged on postoperative day three in good condition. Initial postoperative evaluations, including endoscopic rhinoscopy, showed no signs of local recurrence. The case was discussed in a multidisciplinary tumor board, and adjuvant therapy options, including radiotherapy, were considered. However, as the patient subsequently relocated abroad to continue care, long-term follow-up details are unavailable.

## Discussion

Mucosal melanomas arise in mucous membranes of the body, such as the nasal passages, mouth, throat, vagina, anus, and urinary tract. Mucosal melanomas of the nose and sinuses comprise rare and aggressive tumors with high metastatic rates, which are associated with an unfavorable prognosis [4,6].

Epidemiology

Primary mucosal melanoma accounts for approximately 1.3% of all melanomas, with about 55% occurring in the head and neck region [6]. Within this subset, SNMM is the most common type, yet it remains exceedingly rare-representing less than 1% of all melanomas and under 5% of all sinonasal tumors. These neoplasms originate from melanocytes located in the basal layer of the mucosal epithelium [3]. They mostly affect white individuals between the ages of 50 and 70 years, which is older than the mean age for cutaneous melanomas [7].

Pathophysiology

Multiple mechanisms have been speculated to play a role in the pathophysiology of SNMMs, but the exact pathophysiology still remains unclear [8]. Recent literature suggests several risk factors including smoking, formaldehyde exposure, and chronic inflammation from exposure to certain industrial chemicals, wood dust, pollutants, or toxins [6]. Notably, unlike cutaneous melanomas, mucosal melanomas are not associated with UV radiation or sun exposure [9,10]. Additionally, recent data propose possible immunological mechanisms triggered by microbes, including antigen presentation and activation of the innate and adaptive immunity pathways. Moreover, the biology of mucosal melanomas may differ from cutaneous melanomas, with variations in genetic mutations and molecular pathways, contributing to their aggressive behavior. Genetic profiling has identified recurrent mutations such as NRAS and KIT [10,11].

Prognosis

The prognosis for mucosal melanoma is typically poorer than for its cutaneous counterpart, with a five-year survival of less than 30% and a recurrence rate that exceeds 50% [1,4,5]. This is partly due to the difficulty in early detection and partly due to the more aggressive nature of the tumor itself. Mucosal melanomas may appear in all mucous membranes of the body (nose, gastrointestinal, urinary tract, and reproductive system). Malignancies in these locations typically remain hidden and undetected in early stages, compared to cutaneous cancers, which are more easily noticeable on the skin, delaying early diagnosis and thus complicating surgical management at a more advanced stage [5,12]. The turbinates and the lateral nasal wall are the most commonly affected sites. Less frequently, SNMMs can arise in the paranasal sinuses, with the maxillary sinus being the most typical location, followed by the ethmoid, frontal, and sphenoid sinuses [5,12].

Symptoms

The most common symptoms include epistaxis; breathing difficulties due to unilateral or bilateral nasal congestion or obstruction, which may be mistaken for other conditions such as sinusitis; persistent rhinorrhea, with sometimes blood-streaked nasal discharge; swelling, which may be noticed externally or internally; and nasal and facial pain or numbness. As the disease progresses, more symptoms may appear, such as hyposmia or anosmia, noticeable deformity and nose swelling, and eyesight symptoms such as diplopia and proptosis if the melanoma invades nearby structures such as the eyes or optic nerves [13]. In our case, the 75-year-old patient presented with epistaxis, unilateral nasal congestion, and nasal pain from the last 12 months, which corroborates the above findings.

Diagnosis

Nasal endoscopy is the most definitive diagnostic examination for the detection of NMMs, which typically identifies a polypoid mass that may appear fleshy and either ulcerated or intact, with a dark pigmentation ranging from bluish-black to brown [13]. Diagnosis is confirmed via histopathologic examination.

Histopathology

SNMM is marked by histopathological heterogeneity, featuring melanin pigment, prominent nucleoli, and high mitotic rates. Common cytological forms include plasmacytoid to epithelioid cells, with various morphologies like spindled and rhabdoid types. Histopathological analysis of SNMM should assess features such as cellular morphology, degree of pigmentation, surface ulceration, mitotic activity, extent of necrosis, inflammatory response, perineural or lymphovascular invasion, and infiltration into surrounding tissues, including bone [14]. Accurate histological identification is essential due to SNMM's mimicry of other malignancies and its aggressive nature, highlighted by cases progressing from in situ lesions to invasive melanoma and metastasis despite treatment [15].

Treatment

Treatment options for mucosal melanoma can be more limited and complex due to the tumor's location. Surgical removal is often challenging, and the effectiveness of other treatments, such as radiation and chemotherapy, can vary. The mainstay of treatment for SNMM is surgical resection, with or without the addition of radiotherapy [5]. In recent years, endoscopic techniques have been increasingly adopted, demonstrating survival outcomes comparable to those of traditional open surgery [5]. Achieving negative margins (20 mm) is essential in head and neck oncology, but it can be difficult due to anatomical constraints. While endoscopic visualization has enhanced surgical precision, the complex three-dimensional structure of the region continues to pose challenges to obtaining clear margins. Positive margins and perineural and lymphovascular invasion are all correlated with poor outcomes. According to a 2012 meta-analysis, combining surgery with chemotherapy or immunotherapy may offer a survival benefit for certain patients with SNMM [16]. Radiotherapy is primarily employed in cases of advanced or recurrent disease. A meta-analysis published in 2018 reported that while adjuvant radiotherapy extended overall survival, it did not significantly improve disease-free survival or local disease control [17]. While immunotherapy is well-established in the management of metastatic cutaneous melanoma, its role in treating primary mucosal melanoma is more recent. In the context of SNMM, immunotherapy has been linked to better survival outcomes in patients with distant metastases, though this benefit has not been observed in those without metastatic spread [5]. Although there are no established treatment guidelines, a combination of surgical resection with adjuvant radiotherapy and/or immunotherapy appears to provide the most favorable outcomes. Research into the efficacy of emerging biological therapies is still in progress [5,12].

## Conclusions

Primary malignant melanoma of the nasal cavity is a very uncommon condition that requires careful distinction from other neoplasms of the nasal and paranasal regions. Prompt diagnosis followed by surgical intervention provides the best chance for survival; thus, even minor nasal symptoms should be carefully evaluated. Given the risk of late recurrence, structured postoperative monitoring-typically with endoscopic examinations every three to six months-is strongly recommended.
